# Coronavirus as the Possible Causative Agent of the 1889–1894 Pandemic

**DOI:** 10.3390/idr14030049

**Published:** 2022-06-13

**Authors:** Anton Erkoreka, Josu Hernando-Pérez, Juan Ayllon

**Affiliations:** 1 Basque Museum of the History of Medicine, University of the Basque Country, UPV/EHU, 48940 Leioa, Spain; a.erkoreka@ehu.eus; 2 Grupo de Investigación de Historia Urbana, Población y Patrimonio, University of the Basque Country, UPV/EHU, 48940 Leioa, Spain; 3 Department of Health Sciences, University of Burgos, 09001 Burgos, Spain; jayllon@ubu.es

**Keywords:** 1889–1894 pandemic, coronavirus, HCoV-OC43, influenzavirus A/H1N1, A/H2N2, A/H3N8, Russian flu, history of pandemics

## Abstract

Using new and original nineteenth-century sources, we analysed the epidemiology, clinical features and virology of the 1889 pandemic, which was referred to at the time as ‘Russian flu’ or ‘Asiatic flu’. However, we rejected this identification of the disease as an ‘influenza’, which we believe to have been based on insufficient knowledge of the causative agent and instead posit that the pandemic was caused by a coronavirus. We provide a new account of the 1889–1893 pandemic, with a more detailed chronology that included at least four epidemiological waves. At the end of 1889, a new virus appeared in Europe, which could be identified as the coronavirus HCoV-OC43, causing crude death rates of 1.3 per 1000 population in St Petersburg; 2.1 per 1000 in Paris; 2.8 per 1000 in Bilbao and on the French–Spanish border; between 2.9 and 5.2 per 1000 in small towns in the Basque Country; and 5.8 deaths per 1000 in Madrid, which had the highest death rate. The clinical features of the disease differed from classical influenza pandemics in terms of the latency phase, duration, symptomatology, convalescence, immunity, age and death rates. Another factor to be considered was the neurotropic capacity of the disease. The most frequent form of the 1889 pandemic was the ‘nervous form’, with specific symptoms such as ‘heavy headache’ (*céphalalgie gravative*), tiredness, fever and delirium. There are strong parallels between the 1889–1894 pandemic and the COVID-19 pandemic, and a better understanding of the former may therefore help us to better manage the latter.

## 1. Introduction

Influenza-Like Illness (ILI) is a group of sudden onset symptoms with high fever, chills, malaise, headaches, muscle and joint pain, cough, sore throat, fatigue, mucus, nausea, vomiting, etc., lasting, in mild cases, about one week. The agent responsible is usually a virus—most commonly the influenza virus, which causes seasonal and pandemic flu—but there are also other diseases, such as dengue, COVID-19 and chikungunya, that display similar symptoms.

Historically, the antecedent to pandemic and seasonal types of flu and other such diseases with flu-like syndrome can be seen in the clinical symptoms described in sixteenth- and seventeenth-century treatises with significant names such as ‘*catarrhus epidemicus*’, ‘*tussis epidemicas*’ and ‘*febris catarrhalis epidemicus*’. The great Thomas Sydenham (1624–1689) in his ‘*Opera Medica*’ [1] (written in Latin), devotes an entire chapter to the ‘coughing epidemic’ of 1675, describing perfectly what we would today call a flu syndrome with bacterial superinfection: ‘*Tusses Epidemicae Anni 1675 cum Pleuritide et Peripneumonia Supervenientibus*’. The first book on the subject [2] was published at the end of the eighteenth century. It was written in French and even the title includes both the scientific and common names for the disease: ‘*Historical and reasoned table of catarrhal epidemics, commonly known as* grippe (*influenza*)’. This fascinating work begins by referring to a ‘*fievre catharrale épidémique*’ in 1510 which, the author tells us, was considered by some physicians to be ‘a new disease’: ‘*les Médecins la regarderent comme une maladie nouvelle*’. He adds that doctors referred to this new disease by different names, depending on the symptoms, including ‘*céphalalgie catharrale*’, ‘*toux*’, ‘*catharre’* and ‘*coqueluche*’.

As we can see, from the very first descriptions, the clinical pictures were ill-defined and the aetiology was confused, with the term ‘*grippe*’ being used to designate all of them, regardless of the causative agent. The word ‘*grippe*’ means ‘claw’, and it was used because the disease ‘*grabs*’ its victim, causing an acute sense of extensive blows or breakage. The other term used, namely, ‘influenza’, derived from the fact that the disease was associated with external factors, such as the air, temperature, heavenly bodies and miasmas. From the nineteenth century onwards, this clinical picture came to be generally termed ‘influenza’ in English and Italian, and ‘*grippe*’ in French, German and Spanish. 

The disease referred to as ‘*grippe*’ posed a health problem in the 1830s when European physicians linked it to the cholera pandemic that first struck Europe at that time. Some French doctors of the time insisted that there was an interaction between cholera and influenza. Other leading physicians went so far as to question whether it was really a classifiable illness. Amongst these was Broussais (1772–1838), who claimed that influenza was an ‘invention of people without money and doctors without clients’ (‘*invention des gens sans le sou et des médecins sans clients*’).

The year 1833 saw a major epidemic or pandemic of ‘grippe’, about which several books, pamphlets and articles were published in France [3] and Italy [4]. The pandemic or epidemic of 1837 also sparked an avalanche of publications, some—such as Gohier [5]—offering a good account of the symptoms, while others merely described the evolution of the epidemic [6].

In the mid-nineteenth century, from 1839 to 1889, ‘influenza’ disappeared as a health problem and was not even listed amongst the conditions for which data were collected in the nascent Epidemiological Bulletins [7]. Several proceedings of the *Academie de Medecine de Paris* describe local episodes that lasted, with varying intensity, from autumn to spring. Mortality rates were as high as 1% of the entire affected population. In clinical and symptomatologic terms, these episodes appeared as a combination of flu syndrome, pneumonia, capillary bronchitis, pleurisy and the common cold. 

It was against this backdrop that a major pandemic broke out in November 1889, which first caught the attention of the medical community in Paris. The outbreak initially caused confusion and, in the various countries where it appeared, it was identified by different names, including ‘*grippe*’, ‘*dengue*’, ‘*trancazo*’ and others. In light of the COVID-19 pandemic, we revisited these nineteenth-century epidemics and pandemics with their flu-like symptoms; our goal was to determine whether the conventional interpretation of these episodes is accurate, or whether, based on recent discoveries, we should perhaps reconsider the causative agent and the disease itself. 

In this paper, we clearly postulate that certain coronaviruses made their appearance at the end of the nineteenth century, when they were confused with—and diagnosed as—influenza syndrome. As already stated, the diagnoses of ‘influenza’, ‘catarrh’, ‘dengue’, ‘*coqueluche*’, etc., are highly ambiguous and misleading, in much the same way as ‘pestilence’, ‘plague’ and ‘typhus’ have been historically. We constructed a new account of this 1889 pandemic from a clinical, epidemiological and virological perspective, linking it to the history of coronaviruses [8], especially those affecting humans [9], and using original data from archives, contemporary publications and the information provided by the molecular clock. 

## 2. Methods

In the nineteenth century, France was the world’s most advanced country in the field of medicine, and its archives and libraries are still essential sources for any information on the period. We compiled data from the *Academie de medecine, Archives de Paris*, BIUM and *Archives du services de santé des Armées* (ASSA), all based in Paris; the *Archivio Segreto Vaticano* in the Vatican (Rome); certain religious and civil archives from towns and cities in Spain and France, inter alia Bilbao and Madrid; and from military museums. Finally, we drew on some epidemiological bulletins, such as the *Annuaire statistique de la ville de Paris, Estadistica Demográfica Madrid, Boletín de la Estadística Municipal de Madrid* and the *Boletín Mensual de Estadística Sanitaria de Bilbao*. 

Our information was further complemented using contemporary doctoral theses (Library of the “l’Ancienne Faculté de Médecine” Paris), books, periodicals and articles. PubMed, the medical database of the United States National Library of Medicine, proved essential for comparing these data with the coronavirus and influenza pandemics of the twentieth and twenty-first centuries; there are 93,700 citations for ‘coronavirus’ and 139,300 for ‘influenza’ (last accessed 9 March 2021). From the *Annuaires* and archival material, we built up an extensive database of deaths in Paris and other towns and villages between 1885 and 1894. Using statistical analysis, we defined the different epidemiological waves, and, from population data, we obtained annual, monthly, pathological and age-specific mortality rates. The indicators used were crude mortality rates, excess mortality rates for respiratory pathologies and overall excess mortality rates. These last two indicators were obtained by estimating the number of deaths using data from the previous four years and subtracting this figure from the actual number of deaths in the given year to estimate the mortality attributable to the new virus. This database, created by us, was statistically processed in Stata to obtain the excess mortality and crude death rates.

## 3. Results

In 1888, Paris had a population of 2,326,449, according to the *Annuaire Statistique*. During that year, 51,230 people died in the city, giving an all-cause mortality rate of 22.0 deaths per 1000 population. According to official figures, in the following year, 1889, the mortality rate increased by 1 point to 23.0 deaths per 1000. In 1890, it stood at 22.8 deaths per 1000, and in 1891, it fell back to 21.5 deaths per 1000. 

According to statistics from the *Annuaire*, one of the main causes of death at the end of the nineteenth century was respiratory disease (mainly pneumonia and bronchopneumonia, but we shall also examine acute and chronic bronchitis). Between 1885 and 1889, the main peaks of deaths from these respiratory infections were concentrated in the winter months. The sequence was uniform, with deaths from these causes beginning to increase in the autumn of each year, rising to a peak between January and April and declining sharply in the summer. The peak, therefore, coincided with the coldest months of the year. 

For example, in September 1885, pneumonia accounted for just 200 deaths in Paris, but it rose during that winter, peaking at 682 deaths in March 1886. This represented a 3.4-fold increase in deaths. 

In 1888, the lowest death toll from pneumonia occurred in September when 127 people died of the disease in Paris. That winter, mortality from pneumonia peaked in March at 355 deaths. In other words the number of people dying from pneumonia almost tripled (×2.8). 

### 3.1. Universal Exposition of 1889

The “*Exposition Universelle de Paris”* was staged from 6 May to 31 October 1889, marking the first centenary of the French Revolution. The Eiffel Tower, 312 m high, was built as an imposing entrance arch to the fair; after the exhibition closed, it was decided to keep it, turning it into an icon of the city. At its feet, the exposition occupied a 96-hectare site (237 acres). 

Pavilions were built for the dozens of participating countries, although some monarchical nations—including Germany, the Austro-Hungarian Empire, the United Kingdom, Russia, Spain and Belgium—boycotted the event due to the commemoration of the French Revolution. It attracted some 28 million visitors and thousands of people from all over the world took part, living in the city that year. In keeping with the racist mentality of the time, for example, 400 Africans were showcased to visitors living ‘like savages’ in the ‘*village nègre*’ or ‘*zoo humaine*’; Buffalo Bill Cody’s Wild West Show also took part and there was even a bullring where the best *toreros* of the time fought.

In other words, Paris in 1889 was a hotbed of millions of people from Asia, Africa, America and Europe, who must undoubtedly have brought all kinds of microorganisms to this cosmopolitan city. No sooner had the Exposition closed than a major epidemic broke out, which, within weeks, the health authorities were linking to Russia, calling it the ‘*grippe russe*’ or ‘*grippe asiatique*’. Our research suggested that the time between the emergence of the new disease in Russia and Western Europe was actually very short. Other incongruities include the fact that it appeared earlier in St Petersburg and Paris than in Berlin and Vienna. We should consider the complementary hypothesis that there were two initial foci, one in Paris, associated with the millions of visitors and participants at the *Exposition Universelle*, and another in St Petersburg, a city that had rail links to most of the great capitals of Europe. 

### 3.2. China and St. Petersburg, Russia (November 1889)

Bertillon [10] cites episodes of ‘grippe’ in Canada and Greenland in the spring–summer of 1889. He presumed that the pandemic that affected Europe might have originated in China following the great floods of 1888. Russian sources (the russian researcher Giulietta Meskhidze) point to three areas of what was then the Russian Empire, where serious epidemics were documented in the autumn of 1889. One of these was Tomsk, a major city in the heart of Siberia and one of the stations on the Trans-Siberian railway, as yet uncompleted. What was also under construction at the time was the Transcaspian Railway, whose terminus was in the south-east of the Caspian Sea, in the city of Krasnovodsk (now in Turkmenistan). From this port city, the Moscow rail line passed through Bukhara and the famous Samarkand (now in Uzbekistan), where important epidemics were also documented. Samarkand, whose railway station had opened in 1888, was the most important city on the Silk Road, and was, of course, very well connected to China, from where the new virus could have spread. China—and the heart of Asia in general—have always been viewed as being the origin of many pandemics, including the 1918 influenza outbreak [11]. Another important factor influencing the spread of the disease may have been warfare; the Russian Empire was in the process of completing its military conquest of Central Asia. In 1887, an Anglo-Russian commission agreed on the borderline between the Russian Empire and Afghanistan, and in 1891, the Russians culminated their conquests with the capture of Pamir, a strategic territory bordering China and Afghanistan, and close to the frontier with the British Raj [12]. 

From the south, the pandemic spread to neighbouring Persia. According to Proust [13], it reached Teheran in October but did not extend to the south of the country in the autumn of 1889. From the heart of Asia, the epidemic may have spread to Moscow and St Petersburg. In the latter city, according to local sources, 180,000 people fell ill. In 1890, St Petersburg had a population of 954,400 inhabitants, with a large proportion of foreign residents, especially Germans, who accounted for 5% of the population.

There was a notable increase in the number of deaths in St Petersburg from the third week of November, which could be attributed to the new viral pandemic. The average number of deaths rose from 395 in previous weeks to 604 in the week of 17–23 November and 733 by the last week of the month. The figures remained high throughout the first three weeks of December (634, 626 and 582), but began to fall in the last week of the year (482 deaths), plateauing in January 1890 at an average of 545 deaths per week. 

Using Bertillon’s data, we found that the new pandemic of 1889 led to increased mortality in St Petersburg from 17 November onwards, resulting in an excess mortality rate of 1.3 deaths per 1000 population in the five weeks from mid-November to the end of December. 

### 3.3. Paris, November 1889

The Universal Exposition closed on 31 October. Some days later, on 17 November 1889, Bertillon, Proust and other physicians reported the appearance of a new epidemic disease in Paris, with very mild clinical presentation and no increase in mortality. Historically, official narratives always tended to attribute the source of any pandemic to an outside party, and the 1889 outbreak was no exception. Bertillon speaks of his doubts that it began in St Petersburg (‘*début probable à Saint-Petersbourg*’) on 27 October. As we have already seen, there had been a moderate increase in the death rate in that city in mid-November, from which we could deduce that the new virus was already present in the major capitals of continental Europe by that month. 

All authors dated the start of the pandemic in Paris to 17 November 1889. From 26 November, episodes of the virus affected hundreds of employees in department stores, such as the ‘*Magasin des Nouveautés Le Louvre*’ and other Parisian *grands magasins*—packed as they were with customers from across the globe—as well as public employees, such as those working in the ‘*Direction Générale des Postes et des Télégraphes*’. Most of these were young women and men (young adults). The new epidemic did not lead to a rise in mortality during November; it caused only very mild symptoms, leaving patients ‘incapacitated for work for 4 to 5 days’, according to contemporary sources. In other words, this pandemic had an initial latent phase, with a presentation of very benign cases during the early weeks (mid-November to mid-December 1889). 

### 3.4. Epidemiological Data for Autumn 1889

One hundred and sixty people died of pneumonia in Paris in September 1889, the lowest number of any month that year. Taken together, all the major respiratory causes—pneumonia, bronchopneumonia, acute bronchitis and chronic bronchitis—were responsible for 394 deaths. In November, 194 people died of pneumonia, similar to the figure for the same month in 1888 (218 deaths) and 1887 (244 deaths). That is, although the new virus was already circulating in the city, it was not yet causing serious respiratory complications or deaths. 

In December 1889, the number of deaths from pneumonia soared to 923 (compared with 250 in the same month in 1888 and 218 in 1887), i.e., a 3.9-fold increase. Deaths from all four major respiratory pathologies accounted for 2133 deaths in December 1889 (compared with 739 in 1888 and 656 in 1887), up by a factor of 3.1.

Using these figures we could calculate the mortality rate for the four major respiratory diseases in December 1889 at 0.9 deaths per 1000 population. In the previous year 1888, the rate had been only 0.3 per 1000. Clearly, the new virus was driving up the mortality rate. 

In January 1890, 2345 people died of the four respiratory conditions mentioned (as compared with 863 in 1888 and 1137 in 1887), thus giving a mortality rate from these causes of 1.0 per 1000 population. 

In other words, taking the official data from the *Annuaire*, we calculated a mortality rate of 1.9 deaths per 1000 from respiratory diseases during these six weeks of the epidemic in Paris. A study of excess deaths from all causes, as compared with previous years, gives a figure of 2.1 per 1000 inhabitants, the same as that published by Bertillón.

In February, the number of deaths due to these pathologies declined to similar levels as in previous years: 907 in 1890, as compared with 728 in 1888 and 757 in 1887. By February 1890, we could assume that the first wave of the new pandemic was over.

### 3.5. Maximum Virulence from 15 December 1889 to 31 January 1890

According to Bertillon, three weeks after the outbreak of the epidemic in Paris, around 15 December, the disease became extremely virulent, with mortality rising sharply for approximately six weeks (“*Trois semaines après le debut de l’épidémie, brusquement, la mortalité s’éleva … l’élévation de la mortalité dura environs six semaines*” …), especially increasing the Excess Winter Mortality (EWM). During that period, there were about 5000 more deaths than usual, although doctors certified only 250 cases as being due to ‘*grippe’*. We believe that all of the excess deaths in this period were caused by this new virus classified as ‘*grippe*’.

The new virus spread across continental Europe at extraordinary speed due to the railway network connecting all the major cities; it was also carried by sea to Britain and North America. St Petersburg saw a slight increase in mortality between November 17 and December 21; in Berlin, the death rate almost doubled between 8 December and 11 January; mortality in Vienna increased between 15 December and 11 January; in Paris, the deaths were at their greatest between 15 December and 18 January (although mortality remained high until the end of the month); and in London, the surge lasted from 29 December to 1 February. 

In Paris, according to Vagneron [14], the figures published in the *Annuaire* were hotly disputed by the local press. As a result, two years later, Bertillon drew up a new balance for the pandemic. The epidemiologist estimated the excess mortality attributable to the pandemic between 16 December and 31 January 1890 at exactly 5042 deaths, a figure that was borne out by our estimates. These calculations gave a mortality rate of 2.1 deaths per 1000 population (higher amongst males (2.5‰) than females (1.7‰)).

**Figure 1:**
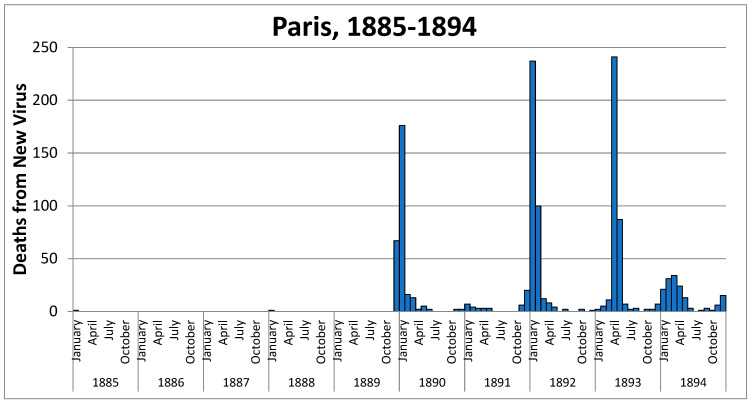
Deaths from the new virus, diagnosed as ‘grippe’, in Paris from 1885 to 1894. Source: “Annuaire statistique de la ville de Paris” [15].

Contemporary health authorities described the Paris epidemic as ‘*grippe*’ and attributed 67 deaths in December 1889 and 176 in January 1890 to this aetiology. There were some doubts about the possibility of dengue, and in Spain and elsewhere, terms such as ‘*trancazo*’ and others were used since physicians did not see it as having the characteristics of classic influenza. Prior to the 1889–1890 pandemic, Parisian records included only a few isolated cases of deaths from ‘*grippe’*: one in January 1885 and another in January 1888. Figure 1 shows the number of cases attributed to this disease.

### 3.6. January–April 1890 Worldwide

In November 1889, the pandemic had spread to every country in Europe between France and Russia; in London, it was well documented in mid-December, and by the end of that month, it was in all of southern Europe from Italy to Portugal. We know that it also spread to North America: Boston and Chicago in December 1889 and San Francisco and Vancouver in January 1890. In South America, the ‘influenza or *trancazo*’ arrived in Buenos Aires and Montevideo in February 1890, in the middle of the summer; it appeared in Algeria and Egypt in January; in January 1890, it passed the Cape of Good Hope and Suez, reaching Bombay in February; Australia and New Zealand in March; and Calcutta in April. That is to say, in the first months of 1890, the new pandemic spread like wildfire, reaching North and South America, Africa, Asia and Oceania. We believe that it was the world’s fastest-spreading pandemic to date [16] (November in Europe; December in North America; January in Africa; February in India and March in Australia). By the summer (August to be precise), it had reached even some of the most remote islands on earth, including Madagascar, Jamaica and Saint Helena. 

### 3.7. Second Wave, 1890–1891

In Europe, the new virus abated in February 1890, although deaths from respiratory causes continued every winter, with a similar incidence. After a summer let-up, rates of respiratory disease began to increase in autumn and cases of the new virus classed as ‘grippe’ were also diagnosed between November 1890 and May 1891. 

**Figure 2:**
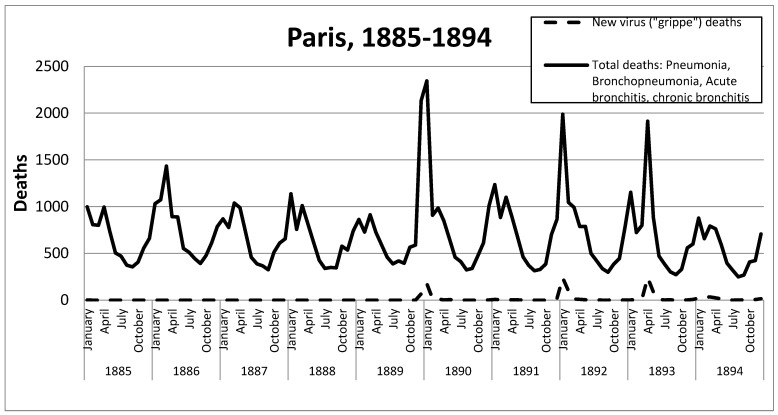
The continuous line shows the deaths from pneumonia, bronchopneumonia, acute bronchitis and chronic bronchitis from 1885 to 1894. The dotted line below shows deaths from the new virus called ‘grippe’. Source: “Annuaire statistique de la ville de Paris” [15].

The month with the highest mortality rate from respiratory diseases was December 1890, with 0.4 deaths per 1000 population. The wave peaked in January 1891, with 1236 deaths, representing a mortality rate of 0.5 per 1000. The numbers dipped and then rose again, forming a twin curve, with the second peak in March, when there were 1100 deaths—a mortality rate of 0.4‰. This camel’s-hump curve, shown in Figure 2, matched that of 1888 and also 1893. The intensity of the second wave was minimal; calculating the excess mortality due to respiratory causes gave a rate of only 0.2 deaths per 1000 population and 0.4 deaths per 1000 for all causes of death. 

### 3.8. Third Wave, 1891–1892

Doctors at the time did not consider the previous peak to be significant. Rather, they attached importance to the re-emergence of the virus at the end of 1891, which they saw as being the last throes of the 1889–1890 pandemic. As we can see in graphs 1 and 2, this epidemic wave was really significant. 

The *Bulletin de l’Academie* [17] included statistics submitted from Persia by Dr Tholozan, referring to this outbreak from November 1891 to January 1892 as the ‘*seconde invasión de la grippe en Perse*’. The clinical picture was very mild. One of the effects of the virus was to leave patients feeling very weak, affecting children and the elderly and causing death, especially amongst people with a prior lung or heart condition. (“*Dans la grande majorité des cas, la maladie n’a duré que quelques jours et a été très légère; malgré cela, elle a laissé après elle una grande faiblesse. Les vieillards et les enfants ont plus souffert que les autres. Beaucoup de décès ont eu lieu sur les personnes atteintes de maladies chroniques des poulmons ou du coeur*”).

In Paris, in the summer of 1891, the number of deaths from respiratory causes fell to a minimum, affecting only 328 people in September. That winter, the pattern of the epidemiological wave of 1889–1890 was repeated. In December 1891, 867 people died of respiratory causes, representing a mortality rate of 0.4 per 1000 inhabitants. Of these deaths, 20 cases were classified as ‘grippe’. This third wave peaked in January, with 1988 deaths due to respiratory pathologies, representing a mortality rate of 0.8 per 1000 inhabitants, including 237 deaths due to ‘grippe’. The figures remained very high in February, with 1045 respiratory deaths (0.4 per 1000), of which, 100 cases were registered as ‘*grippe*’. From that month on, both viral and bacterial infections declined to similar levels as in previous years.

In total, the cumulative mortality rate for respiratory pathologies over these three months was 1.6 per 1000. As we did for previous waves, we examined the excess mortality and found a figure of 0.6 per 1000 for respiratory pathologies and 0.8 per 1000 for all causes of death. That is to say, the strong resurgence of the new virus during the winter of 1891–1892 clearly drove up the number of respiratory overinfections without reaching the severity of the first pandemic peak of 1889–1890.

### 3.9. Fourth Wave, 1893

The number of deaths due to respiratory pathologies fell again at the end of the summer of 1892 and began to increase in the autumn, reaching a peak in January 1893 similar to those of the years prior to 1889, with a rate of 0.4 deaths per 1000 population. However, from April onwards, there was a second notable increase in mortality from these conditions, coinciding with the maximum number of deaths diagnosed as ‘*grippe*’, which reached 241 that month. It is important to note that on this occasion, the new virus appeared three months later than in the two waves of 1889–1890 and 1891–1892 and was the trigger for the increase in respiratory pathologies. 

In April, 1914 people died of respiratory illnesses, with a mortality rate of 0.6 per 1000. This was more than twice the average number of deaths (869) from that cause during the same month in the years since 1885. In May, the number of deaths from respiratory disease plateaued at 884 (0.4 per 1000), which was still higher than the average for the same month in previous years. Deaths diagnosed as being caused by ‘grippe’ also fell that May to 87 and, from then on, began to be residual, with deaths due to respiratory pathologies returning to the annual minimums. 

The cumulative crude mortality rate in these two months for respiratory diseases was 1 per 1000 inhabitants (0.6 + 0.4). Turning again to excess mortality, we found an increase in deaths from all causes of death of 0.7 per 1000 during this period and 0.5 per 1000 from respiratory diseases alone.

In the summer of 1893, the number of deaths again fell to a minimum, only to rise once more in the first months of 1894. The highest number of deaths due to respiratory diseases came in January at 878 (0.3 per 1000). This figure and the pattern on the graph were similar to those seen in years before 1889 and we must therefore presume that the cycle that had begun in 1889 came to an end in 1894. The *Annuaire* listed a few cases (123 to be precise) diagnosed as ‘grippe’, most between January and May 1894. This would therefore appear to mark the end of the pandemic caused by the new virus.

### 3.10. Summary of Mortality in Paris

From a demographic and epidemiological perspective, by the end of the nineteenth century, Paris had got its mortality figures significantly under control. It was one of the most advanced cities in the world at addressing the demographic transition (reduction in mortality, birth rate and increase in life expectancy at birth) (Vallin, Meslé [18], Lee [19]). In 1888, it had a crude mortality rate of 22.0 deaths per 1000 population. The 7673 deaths from all causes in December 1889 and the 7367 deaths from all causes in January 1890 gave all-cause mortality rates for those two months of 3.2 and 3.0 deaths per 1000 inhabitants respectively, the city’s highest figures in the last decade of the nineteenth century. From then on, monthly mortality rates rarely exceeded 2 per 1000. 

The table (Table 1) below offers a summary of the four epidemic waves that we attributed to the new virus that first appeared in autumn 1889. The first column shows the excess deaths from all causes and their corresponding Crude Death Rates (CDRs) per 1000 inhabitants. The second column shows excess deaths due solely to respiratory causes (pneumonia, bronchopneumonia, acute bronchitis, chronic bronchitis and influenza). In both cases, we subtracted the average number of deaths from the same cause from the corresponding absolute number during the same period (1885 to 1888). Summing up, we could say that in the four waves that could be attributed to the new virus between 1889 and 1893, it caused a mortality rate of 3.9 deaths per 1000 inhabitants. 

### 3.11. The Pandemic on the French–Spanish Border and in Madrid

Bertillon and Proust included maps of Europe and France showing the distribution of the pandemic. In France, the pandemic spread south from Paris (peaking from 5 December to 23 January), reaching Bordeaux (from 1 to 24 January) and Toulouse (from 2 to 30 January). The railway lines linking Paris and Madrid converged in Irun, where we consulted parish registers to study the deaths caused by this pandemic. All deaths from the new virus were diagnosed as ‘pneumonia’. The first victim was a 78-year-old woman who died on December 28 and the last was a 66-year-old man who died on February 7. The mortality rate in the town, for those diagnosed with pneumonia alone, was 2.8 deaths per 1000 population. The mortality rate from this cause in other Basque towns—such as Gernika and Durango—was higher, reaching 4.9 and 5.2 deaths per 1000, respectively, in those six weeks. The epidemic did not appear in other towns we studied in the north of Navarre, such as Etxarri-Aranatz and Sangüesa. 

Using the civil register, we also calculated the excess mortality in the city of Bilbao (population 55,205 in 1890), where industrialization was beginning to take off. In December, there was a slight increase in excess mortality of 0.2 per 1000 inhabitants. The main peak in deaths occurred in January, with mortality attributable to the new virus of 2.6 per 1000 population. This gave us an accumulated excess mortality rate of 2.8 deaths per 1000 population in Bilbao, somewhat higher than the 2.1 deaths per 1000 inhabitants in Paris. 

In Madrid (1887 population: 470,283), the new pathology was referred to first as ‘*trancazo*’ and later as ‘*grippe*’, although there were persistent suggestions that it might be ‘dengue’. Whatever the diagnosis, the new virus caused a notable increase in mortality: during the first wave (December 1889–January 1890), according to Ramiro et al. [20], there was excess mortality from all causes of 5.8 deaths per 1000 inhabitants.

One of the reasons for this high figure in Madrid was that the city was considerably behind Paris in the process of demographic transition. Whereas in the French capital, the annual crude mortality rates for the 1890s stood at around 20 deaths per 1000 population, in Spain, the figure was 31 per 1000 inhabitants [21]. In Madrid, the regular mortality rate was even higher [22] at 41 per 1000 according to García. This astronomical figure was a result of the city’s demographic phase, its deficient hygienic conditions, overcrowding, the sanitary structure, food and other factors as yet unidentified. This is a clear case of what Reher [23] calls the ‘urban penalty’ as compared to the rural milieu.

As we have seen, the impact of the 1889 pandemic varied significantly from one end of Europe to the other. From St Petersburg to Madrid, mortality rates ranged, according to our calculations, from 1.3 to 5.8 per 1000 population. Valleron et al. [24] estimated a total global death toll of one million. Mortality rates published by Valtat et al. [25] ranged from 1 to 2.8 deaths per 1000 population. 

### 3.12. Age of the Deceased

**Figure 3:**
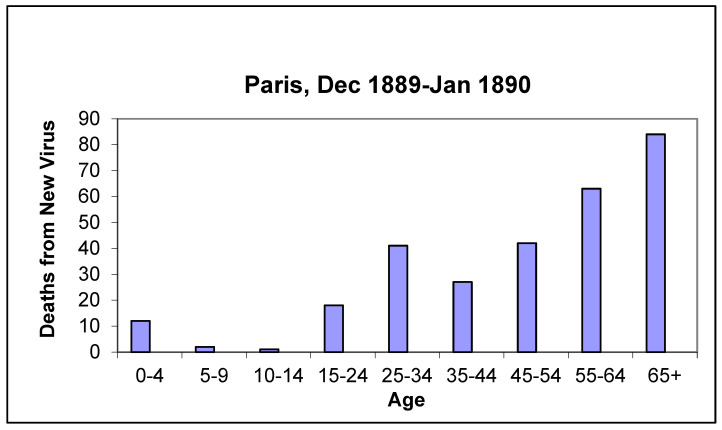
Ages of those who died of the new virus, diagnosed as ‘grippe’, in Paris from December 1889 to January 1890. Source: “Annuaire statistique de la ville de Paris” [15].

**Figure 4:**
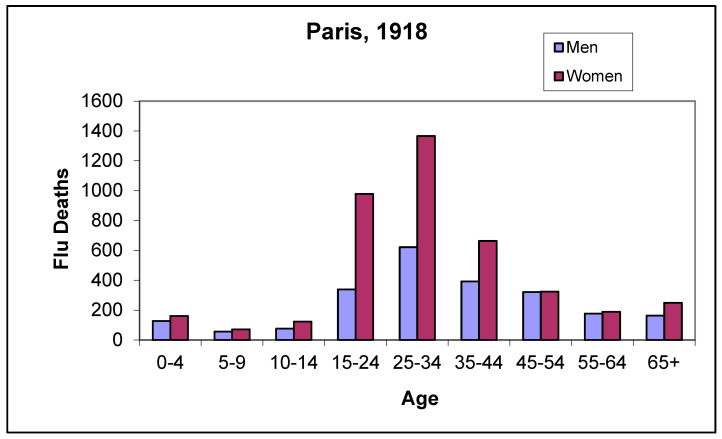
Ages of those dying of influenza (A/H1N1 virus) in Paris in 1918. Source: “Annuaire statistique de la ville de Paris” [15].

Figure 3 shows the distribution of deaths in Paris due to the new virus. Almost half (44.9%) were aged over 55, whereas in the 1918 influenza pandemic, this age group accounted for only 12.1% of the total, a much lower figure [26]. Young adults (aged 15–44) accounted for 34.7% of all deaths. This was the major difference with the Spanish influenza pandemic in that young adults (aged between 15 and 44) accounted for 68.2% of all deaths in 1918 (Figure 4). In other words, twice as many young adults died in the 1918 Spanish flu epidemic than in the 1889–1890 pandemic. 

These age differences were all the more remarkable given that *fin-de-siecle* Paris was a young society with high birth rates and a population aged mostly under 30. There were far fewer over-55s than young people, and yet the deaths of 1889–1890 were concentrated in this older age group. This different impact by age became even more obvious when we compared the number of deaths to the number of individuals in each age group. Bertillon made this calculation for the first wave of the pandemic and the differences were highly significant: the CDR in this pandemic was directly proportional to age. Mortality rates remained consistently below 0.5 per 1000 population in population groups aged under 20. Amongst those aged over 55, the rates were above 5.7 per 1000 for males and 3.4 per 1000 for females. For the 70+ age group, mortality rates were in excess of 11 per 1000 amongst both sexes. Finally, amongst the over-80s, the CDR was close to 30 per 1000. These data confirmed the significant difference in impact by age in the pandemic in Paris.

In Irun on the French–Spanish border, over-55s accounted for 44.4% of all deaths from the new virus, an identical proportion to that seen in Paris, whereas young adults (aged 15–44) represented only 14.8%. Young children accounted for 22.2% of total deaths as a result of the measles and diphtheria epidemics that struck the Basque Country in the closing months of 1889. 

This mortality pattern in the 1889 pandemic, affecting the oldest cohorts most, was mirrored in Madrid (Ramiro et al.) and other European capitals (Valtat et al.). 

**Figure 5:**
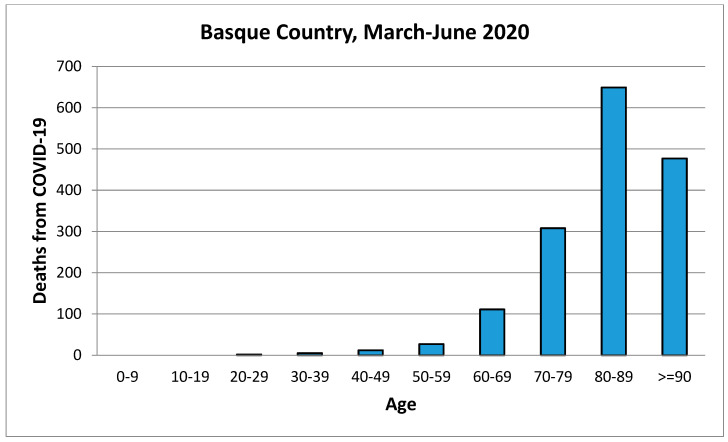
Ages of those who died during the first wave of COVID-19, in Basque Country. Source: A. Erkoreka [16].

When comparing this data with the graphs of deaths by age in the first wave of COVID-19 (Figure 5), we found that it was a very similar model. Deaths were concentrated in age groups over 60 years, with the largest group of deceased between 80–89 years old [16].

### 3.13. Clinical Features

When the 1889 epidemic first began, many physicians were puzzled by the clinical picture, which varied greatly from that of classical influenza. The last major epidemic had taken place in 1837 and was characterized mainly by respiratory symptoms. A doctoral thesis of the time [5] stated categorically that the 1837 influenza ‘almost universally presents in the form of bronchitis’, “*la grippe se presente dans la presque universalité des cas sous la forme d’une bronchite*”, even adding that it would have been more apt to call it ‘epidemic bronchitis’.

Likewise, when the 1918 influenza pandemic appeared, many authors noted the enormous differences between it and the 1889 outbreak. Using Widal’s description of the 1889 pandemic in Charcot’s *Treatise on Medicine* [27], together with that of Piga and Lamas [28] and other authors who studied the 1918 pandemic, let us now examine some notable differences between the 1918 and 1889 pandemics. We know that the causative agent in 1918 was the A/H1N1 influenza virus, and we believe the 1889 influenza virus had many characteristics of the coronaviruses responsible for the 2020 pandemic.

- -Latency phase: In November 1889, the virus was already present in Paris and other European cities, where it caused a very mild clinical picture, with similar characteristics to a common cold. In this regard, it is worth remembering that, although the first cases of COVID-19 in Europe were officially identified in February 2020, the coronavirus appeared to have been circulating on the continent since the end of 2019. In other words, there was also a latency period or phase before the outbreak of the pandemic proper [29].
- -Duration: The epidemic peak of the 1889–1890 pandemic lasted 6 weeks in each of the cities affected. In the autumn of 1918, the peak in the towns and cities we studied in Western Europe lasted almost four weeks.
- Miscellaneous signs and symptoms: Patients in the 1889–1890 pandemic experienced abundant expectoration, which was rarely haemorrhagic and there was no bleeding elsewhere in the body. In 1918, in contrast, there was little expectoration, and it was frequently haemorrhagic. Bleeding was also frequent in other parts of the body.
- Symptomatology: Symptoms in 1889 were mainly neurological, i.e., of the nervous system, whereas in 1918, the most significant symptomatology was in the respiratory system, together with other general symptoms.
- Duration of the clinical process: Patients took much longer to recover from infection in the 1889 pandemic than in the 1918 pandemic. The same is true for COVID-19.
- Mortality rate: The CDR of the 1889 pandemic in Europe averaged about 2 deaths per 1000 population. This was much lower than the mortality rate for the so-called Spanish flu, at 11 deaths per 1000 in Europe and up to 25 deaths per 1000 in the global population as a whole.
- Age: The 1889 pandemic resulted in much higher mortality amongst the elderly. In Paris, 44.9% of those who died of influenza in December 1889 and January 1890 were aged over 55. In contrast, only 12.1% of those who died of influenza in Paris in 1918 belonged to this age group. We have already seen that the exact opposite was the case amongst young adults, as Figure 4 shows.
- Sex: In the 1889 pandemic more men died than women; Bertillon gives a figure of 2.5 deaths per 1000 amongst men and 1.7 per 1000 amongst women, 59.5% of the deceased were men and 40.5% were women. In the 1918 pandemic, according to data from the population sample studied by Erkoreka [30], the difference was far less pronounced: 53.0%/47.0% of deaths with a diagnosis of influenza and 51.6%/48.4% of those dying from respiratory pathologies. In 2020 the proportion of men dying from COVID-19 has also been much higher, with the two age groups most affected by the pandemic being those aged 80–89 and those aged over 90.
- Immunity: The 1889 pandemic produced little immunity, while the 1918 pandemic did.
- Convalescence and after-effects: Amongst patients in 1889, convalescence was long and tedious. In contrast, amongst the patients of 1918, it was much shorter. What in 2020 and 2021 we call long-covid or post-acute COVID-19 was perfectly mirrored by a commentary in Charcot’s *Treatise on Medicine* (published in 1892) when he states that in the 1889–1890 pandemic, patients were left with ‘fatigue and a very marked depression of strength, prostration, dizziness, anorexia, vomiting … There usually persists a neurasthenic state from which many of the former flu victims have yet to recover at the time of writing, that is, more than a year after the epidemic’.

### 3.14. Nervous Influenza (‘Grippe Nerveuse’)

The best study of the 1889 pandemic is that published by Proust in the *Bulletin de l’Académie de médecine* in 1892, which stresses that the main form of presentation was ‘nervous influenza’ (*grippe nerveuse)* [13]: ‘Extremely sudden onset; very severe headache; very sharp pain in the eye sockets; crushing sensation of the eyes; very pronounced arthralgic and muscular pains … in two or three days, these symptoms cease and a gasping (whooping) cough appears, without expectoration; extreme weakness … rash, eruptions, hives … these symptoms indicate a general neural disorder rather than a state of clear inflammation’ (“*Les divers symptômes observées dans la grippe révèlent bien plutôt un trouble nerveux général, qu’un état d’inflamation franche*”).

In the last decade of the nineteenth century and the first years of the twentieth, a large number of doctoral theses and articles were published in France stressing the multiple nervous and psychic alterations caused by the 1889 pandemic. Trastour [31] remarked that ‘in a large number of cases of influenza, neural and cerebral phenomena predominated; in some cases these phenomena were the only symptoms of the disease. (…) In all cases a dejection and extraordinary weakness are observed … this state persists for a long time, even after the other symptoms in the lungs, and stomach and febrile phenomena have disappeared’. ‘The disease comes on suddenly with a severe headache, general tiredness and fever. Patient takes to their beds and the delirium appears; this may be temporary or manifest itself only at night, although in some cases it persists for weeks uninterruptedly, day and night. The patient may also fall into a state of drowsiness and dullness …’. This description mirrors the symptoms observed in 2020 and 2021. With regard to mental illness, a work by Leledy [32]—endorsed by Charcot—stated that the 1889 pandemic caused depression, delirium, psychosis and other serious psychiatric conditions. 

It is worth drawing attention to the ‘heavy headache’ *(céphalalgie gravative)* that Proust cites as being another of the main symptoms of the 1889 pandemic, saying that this type of headache and cough persisted after the other symptoms had ceased and the disease itself had ended (“*La céphalalgie gravative, l’affaiblissement remarquable que ainsi que la toux, persistait après la cessation des autres sypmtômes, la terminaison favorable de la maladie*”).

Based on all these symptoms, it is clear that the virus responsible for the 1889 pandemic was different from that which caused the 1837 and 1918 pandemics. The well-documented presence of neural symptoms led us to consider an aetiological agent with a neurotropic capacity or a response by the system to the infection that negatively affected the central nervous system, or perhaps a combination of the two, as we see in the case of COVID-19. The 1889 pandemic and the SARS-CoV-2 pandemic of 2020 share many elements in common and have some important parallels, which led us to posit that the agent responsible for both pandemics may have been a coronavirus. 

### 3.15. Aetiology of the Pandemic. Serological and Molecular Indications

The great influenza pandemic of 1918 left a strong mark on the birth of virology and epidemiology in the twentieth and twenty-first centuries and the surveillance measures and contingency plans put in place across the world. Indeed, up until the outbreak of COVID-19, the WHO’s definition of pandemic referred exclusively to influenza [33]. It is hardly surprising that, in the ever difficult search for the pathogens behind historical illnesses, influenza is very often one of the usual suspects, especially if the symptomatology includes a respiratory infection and the wide range of symptoms we know as ‘Influenza-Like Illness’ (‘flu-like syndrome’). The few virologists and epidemiologists who have so far studied the 1889 pandemic have not questioned whether it was influenza, limiting themselves to identifying the type of influenza concerned.

Originally, the aetiological agent of the 1918 pandemic was determined to be A/H1N1 influenza in the 1930s, when the isolation of the first influenza viruses with that serotype allowed for the identification of high titers of antibodies in people who had been exposed to the pandemic [34]. This was subsequently validated by the retrieval and characterization of sequences from the virus [35]. This same trend in sero-archaeology (reviewed by Dowles) [36] is what initially led writers to identify the causative agent of the 1889–90 influenza as being an influenza virus of subtype H2 (after the A/H2N2 pandemic of 1957) and, more recently, as an A/H3 virus, with the latter being the most widely accepted hypothesis at this time. The identity of the 1889–90 pathogen as a type A/H3NX influenza (probably A/H3N8) [37] is supported both by these serological analyses and by the pattern of mortality observed during the 1968 pandemic when the introduction of a new type A/H3N2 virus did not cause excess mortality amongst the older population.

The data pointing to A/H3NX, while interesting, cannot be considered conclusive for several reasons. First, sero-archaeology is far from being an exact science, relying on highly variable tests, such as haemagglutination inhibition (HI), especially when it comes to drawing conclusions about immunological events that are decades apart. Second, the H3NX hypothesis identifies the proven circulation of a virus (A/H3N2) with the causation of a pandemic because of the widely established belief throughout the twentieth century that the introduction of any new form of influenza would cause a major upset. However, the very limited impact of the 2009 pandemic (and arguably that of the reintroduction of influenza A/H1N1 in 1977) demonstrated that the emergence of new serotypes may have gone largely unnoticed, especially in the troubled epidemiological context of the nineteenth century. In short, this hypothesis is based on extrapolations restricted by only having samples of three well-studied pandemic events (1918, 1957 and 1968). The 2009 pandemic significantly altered our perception of pandemics, and in the wake of COVID-19, we may need to revisit the paradigm. 

The new context in which we find ourselves in 2021, and the increased focus on coronaviruses as the new star in the firmament of emerging diseases, have heightened interest in an article published in 2005 by Vijgen [38], Van Ranst et al. For the first time, the article showed the complete genome sequence of OC43 (HCoV-OC43), one of four coronaviruses causing the common cold in humans. OC43 is part of the genus *Betacoronavirus*, which also includes the subgenus Sarbecovirus to which MERS-CoV, SARS-CoV and SARS-CoV-2 all belong. The study by Van Ranst et al. offers a phylogenetic analysis of the OC43 genome and establishes the most probable date for separation of its lineage from that of its closely related strain, namely, bovine coronavirus (BCoV). This process, thoroughly tested by the authors with three different datation methods, consistently places the date of the emergence at around 1890.

The authors argued that the new virus was introduced to humans from animals and suggested the possibility that OC43 was the aetiological agent of the so-called ‘*Russian flu*’ of 1889–1890. In addition to the coincidence in time, the well-established neurotropism of OC43 (MacIntosh et al. [39], Desforges et al. [40]) would be consistent with the multiple nervous and psychic alterations caused by the virus responsible for the 1889 pandemic, as we have just seen. The clinical picture described by physicians at the end of the nineteenth century, which even distinguishes a ‘nervous form’ of presentation of the 1889 pandemic and specific symptoms, such as ‘severe headache’, all tantalisingly point to the possibility that OC43 was the causative agent of the 1889 pandemic.

## 4. Discussion

During these recent months, the historical and medical interest in the study of this historic pandemic has increased [41,42]. 

In any case, given the enormous advances of recent years in the field of archaeogenetics, with a growing collection of genetic information on former pathogens being obtained from different sources [43], it might be worthwhile considering a study of samples preserved from patients who died in the 1889–90 pandemic. Data of sufficient quality could be obtained from them to shed new light on the identity of the aetiological agent. Any such study will benefit from the existence of a short list of likely suspects, including the HCoV-OC43 coronavirus and influenza virus subtypes H3 and H2, for which specific and more sensitive searches can be conducted. 

The causative agent will have to be identified in biological samples from the end of the nineteenth century, such as the organs and preparations kept in the Basque Museum of the History of Medicine [44]. We possess a collection of 400 human organs and biological materials, preserved using the Kaiserling formula. Together with samples from other collections, these may allow us to retrieve and sequence the virus responsible for the 1889 pandemic. This task remains for the future.

## 5. Conclusions

The COVID-19 pandemic that started in 2020 has forced us to revise our perception of past pandemics. Principally the one that began in 1889, which was the first of the great modern viral pandemics that spread like wildfire throughout the world in record time. That pandemic of 1889 was baptized as “Russian flu” since influenza or “*grippe*” was the symptomatology that most resembled it, although its etiological agent was unknown.

After investigating the history, epidemiology, the symptomatology and virology of the 1889 pandemic, we propose a new narrative involving at least four epidemiological waves between 1889 and 1894; a mortality rate of around 2.1 deaths per 1000 inhabitants for the first wave (the highly reliable figure for Paris can be extrapolated to other cities and countries); and a the symptomatology closer to that of COVID-19 than to the classical pandemics of 1837 and 1918. For all these reasons, we hypothesize that the causal agent of the 1889 pandemic was a coronavirus, probably HCoV-OC43, which following its sudden and aggressive appearance gradually lost its virulence over the years to simply become one of the four coronaviruses that cause common colds in humans. If history repeats itself, this could also be the fate of SARS-CoV-2 in coming years.

## Figures and Tables

**Table 1:** Excess mortality from all causes and from respiratory pathologies in Paris between 1889 and 1893. Source: “Annuaire statistique de la ville de Paris” [15].

Waves	Excess Deaths from All Causes	Excess Mortality Rate, Total per 1000	Excess Deaths Due to Infectious Respiratory Pathologies	Excess Mortality Rate, Respiratory System per 1000
First wave (December 1889–January 1890)	4953	2.1	2793	1.2
Second wave (December 1890–January 1891)	859	0.4	562	0.2
Third wave (December 1891–February 1892)	1887	0.8	1348	0.6
Fourth wave (April–May 1893)	1719	0.7	1204	0.5
Total	9418	3.9	5907	2.5

## Data Availability

Not applicable.
